# Self-assembled micro and nano rod-shaped porphyrin@Bi_12_O_17_Cl_2_ composite as an efficient photocatalyst for degradation of organic contaminants

**DOI:** 10.1186/s11671-023-03915-4

**Published:** 2023-10-31

**Authors:** Osemeikhian Ogbeifun, Shepherd M. Tichapondwa, Evans M. N. Chirwa

**Affiliations:** Water Utilization and Environmental Engineering Division, Department of Chemical Engineering, University of Pretoria, Pretoria, 0002 South Africa

**Keywords:** Bi_12_O_17_Cl_2_, Photosensitizer, Charge recombination, Heterostructure, Rod-shaped aggregated porphyrin

## Abstract

Bi_12_O_17_Cl_2_ is a potential photocatalyst in practical applications due to its excellent photostability, visible light activity, and competitive bandgap energy. However, the fast recombination of photogenerated charge carriers makes it impractical for pollution mitigation. Recently, aggregated porphyrins have emerged as photosensitizers in light-dependent applications such as photocatalysis. Although Bi_12_O_17_Cl_2_ and porphyrin can function as separate photocatalysts, their photocatalytic properties in terms of visible light adsorption, charge separation and transport, can be improved when they are combined to form heterostructure. In this study, rod-shaped aggregated 5,10,15, 20-Tetrakis (4-carboxyphenyl) porphyrin was synthesized by CTAB-assisted, self-assembly strategy and Bi_12_O_17_Cl_2_ by a facile microwave method. The porphyrin and Bi_12_O_17_Cl_2_ were combined to generate a series of *x%*Porphyrin@Bi_12_O_17_Cl_2_ having 0.02% wt., 0.1% wt., 0.4% wt., 1% wt. and 10% wt. as compositions of porphyrin. The materials’ photocatalytic degradation efficiency was tested on Rhodamine B dye as a representative pollutant. The best and worst performances were reported for 1*%*Porphyrin@Bi_12_O_17_Cl_2_ and 10*%*Porphyrin@Bi_12_O_17_Cl_2_, respectively, which are 3.1 and 0.5 times increases in efficiency compared to pure Bi_12_O_17_Cl_2_. From the radical trapping experiment, electrons and superoxide were the dominant reactive species in the degradation process. The enhanced photocatalytic capability of the materials was attributed to the photosensitizing property of porphyrin and the heterojunction formation, which promotes the separation of photogenerated charge carriers. A plausible step-scheme (S-scheme) was proposed for the photocatalytic degradation mechanism. The S-scheme provided the high redox potential of the photogenerated charge carriers. The findings herein offer a new option for improving the photocatalytic performance of Bi_12_O_17_Cl_2_ for environmental applications through the photosensitization strategy.

**Graphical abstract:**

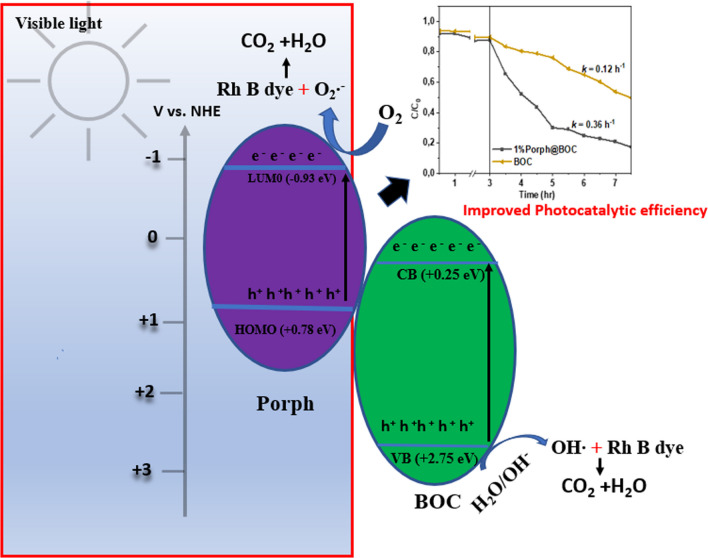

## Introduction

Since the introduction of bismuth oxyhalides (BiOX, where X = F, Cl, Br, or I) as promising photocatalysts for mitigating environmental contamination, the focus has been on improving the performance of the material. This endeavour led to the synthesis and application of bismuth-rich complex oxyhalides (Bi_x_O_y_X_z_) photocatalyst material [1]. Bi_12_O_17_Cl_2_ is a notable member of this complex oxyhalide family [2], possessing excellent, adjustable bandgap energy [3], and visible light or infrared activity [4], a benign/non-toxic nature [5]. However, the quick recombination of photogenerated charge carriers (e^−^ and h^+^) as they are produced limits the capacity of the material to oxidize target substrates [6].

**Scheme 1:**
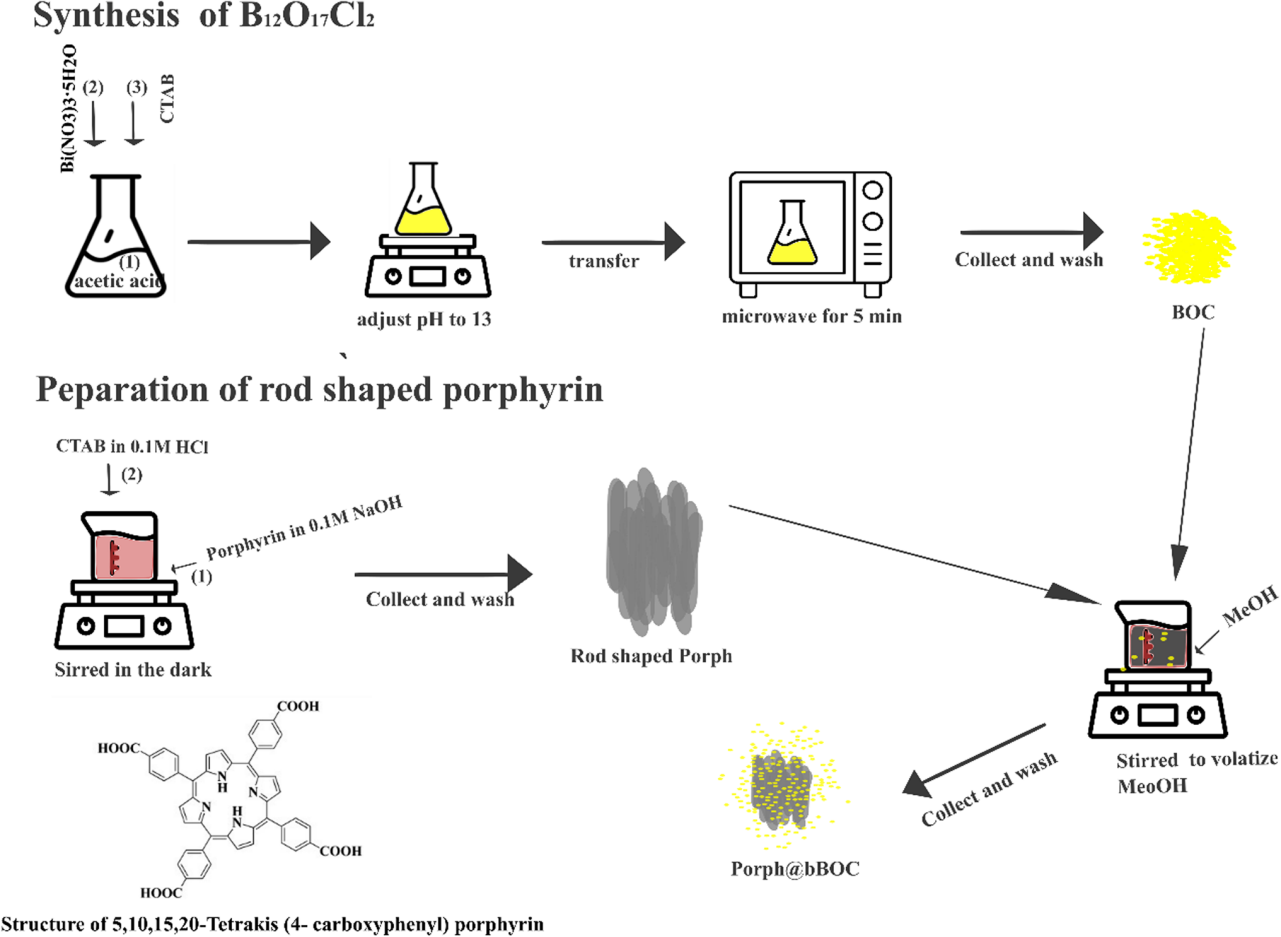
Synthesis of aggregated porphyrin, Bi_12_O_17_Cl_2_ and Porph@BOC and structure of 5,10,15,20-Tetrakis (4- carboxyphenyl) porphyrin

To enhance the photocatalytic degradation ability of Bi_12_O_17_Cl_2,_ various strategies have been employed, including heterojunction fabrication [7], ion doping [8], selective synthesis routes [9], plasmon resonance effect [10], solid solution [11] and photosensitization effect [12]. The photosensitization strategy involves applying a photosensitizer such a porphyrin, to photocatalyst of interest to increase visible light utilization in the resulting composite heterostructure [13]. Porphyrin is a group of organic molecules made of four pyrrole rings that are linked with methine bridges to form a planer macrocyclic structure known as porphyrin ring [14] (Scheme 1). They can be coordinated with metals such as magnesium, iron, copper, cobalt, and zinc at the center of the ring to yield metalloporphyrin [15]. In photocatalysis or other light-harvesting applications, porphyrin is either used alone or immobilized on inorganic photocatalysts to form organic–inorganic systems. Porphyrin strongly absorbs visible light, producing a photochemical reaction [16], that generates triplet (activated state) that can oxidize contaminants in the medium [17].

Porphyrin can assume various nano-/micro-assemblies and morphologies such as nanorods, nanobelts, nanotubes, and nanofiber bundles, hollow tubes, rods, fibre bundles, hollow hexagonal nano-prisms, sheets, solid spheres, hollow spheres and so forth [18]. Two types of molecular aggregation occur in porphyrin, namely J- and H- type aggregates which are assembled through various interactions such as hydrogen bonding, π-π stacking, hydrophobic and electrostatic interactions and van der Waals forces [19]. Typically, these interactions are triggered by mixing acidic solution of porphyrin with basic surfactant solution. The deprotonation-protonation reactions in the acid and subsequent neutralization lead to insoluble porphyrin and promote the confined self-assembly process within the hydrophobic cores of surfactant micelles [20]. The J-aggregation is described as offset-stack, step-like, edge-to-edge, and the H-aggregation as face-to-face arrangements [21]. The high structural order and rigid framework of the aggregates provide unique electronic and spectroscopic properties, resistance to attack by reactive species and photostability[22]. Aggregated porphyrins are comparable to the structure of chlorophyll, where the molecules are arranged in array, promoting charge separation better than their monomeric form [23]. In certain instance, aggregated porphyrin supramolecules are preferred in terms of light trapping, electron donation, electron accepting, and transport [24]. Self-assembled nano- and micro-porphyrins find applications in supramolecular chemistry, light harvesting, photosensitization, photonics, and sensor technologies [18].

The morphology of the aggregated porphyrin molecules influences their photocatalytic activity. Mandal and co-authors reported a photocatalytic degradation of Rhodamine B dye (RhB) of 56%, 81%, 79%, and 71% over spherical, rod, flakes, and flower-shaped Tetrakis (4-carboxyphenyl) porphyrin, respectively [25]. It was reported that the rod-shaped aggregate structure possesses the highest photocatalytic activity amongst other morphologies studied [26]. While the flake- and flower-shaped aggregates were characterized to be in the J- and H-aggregation forms, the rods exist only in the J-aggregation form. The J-aggregation form enhances the coherent electronic delocalization for long-lived charge carriers, making it a more efficient photocatalyst [27].

When porphyrin is irradiated, excited electrons move from highest occupied molecular orbital (HOMO) to the lowest unoccupied molecular orbital (LUMO) [28]. The LUMO electrons are transferred and inject into the conduction band (CB) of the host material [29], separating them from the holes in the system [30], thereby boosting the photocatalytic performance. Porphyrin has been applied to various materials such as graphite carbon nitrides [31]; Fe_3_O_4_ [32]; TiO_2_ [29], BiOCl [33], ZnO nanofiber  [34], graphene [35]. Several studies on the influence and interaction of porphyrin with TiO_2_ have been widely researched considering the prominence of TiO_2_ as a pioneer material in photocatalysis [22]. However, few studies have investigated the porphyrin-Bi_x_O_y_X_z_, system, despite the diverse nature of porphyrin structures and morphologies available, which holds potential in the field of photocatalysis. Furthermore, there is a discrepancy in the reported optimum porphyrin content in the porphyrin-Bi_x_O_y_X_z_ composite. Huang et al. and Zhao et al. reported an optimum porphyrin content of 1% wt. and 2% wt., respectively, in porphyrin-BiOCl composite, while Wang et al. reported an optimum porphyrin content of 0.5% wt. in porphyrin-Bi_12_O_17_Cl_2_ composite. It is likely that the morphology of porphyrins and the type of bismuth oxyhalides involved are factors to be considered in this regard.

In this study, self-assembled rod-shaped 5,10,15, 20-Tetrakis (4-carboxyphenyl) porphyrin was prepared and anchored to Bi_12_O_17_Cl_2_. The photocatalytic activity of the surface modified-Bi_12_O_17_Cl_2_ was evaluated by measuring the degradation of RhB dye under visible light. The optimum composition of porphyrin in the composite and their photocatalytic efficiency was determined. Furthermore, charge carrier separation, transport, redox potential, stability and possible mechanism of degradation on the material were proposed.

## Experiment

### Materials and method

#### Material

Bismuth (III) nitrate pentahydrate (Bi(NO_3_)_3_·5H_2_O), cetyltrimethyl ammonium chloride (CTAC), cetyltrimethyl ammonium bromide (CTAB) (obtained from Sigma Aldrich, South Africa), ethanol, glacial acetic acid, NaOH, rhodamine B (RhB) dye disodium ethylenediaminetetraacetate dihydrate (EDTA-2Na), 1,4-benzoquinone (BQ), isopropanol alcohol (IPA), Silver nitrate (AgNO_3_) (obtained from Glass World Pty, South Africa), 5,10,15,20-Tetrakis (4-carboxyphenyl) porphyrin (obtained from PorphyChem, France) were used in this study. All reagents were used as received from suppliers and deionized water was used throughout the experiments.

#### Synthesis of Bi_12_O_17_Cl_2_

Scheme 1 depicts the synthesis of aggregated porphyrin (Porph), Bi_12_O_17_Cl_2_ (BOC) and Porph@BOC. In brief, hierarchical BOC  was synthesized by dehalogenation of BiOCl precipitate in alkaline medium at pH 13. The BOC precipitate was microwave-treated to complete the crystallization process. Pure BOC was synthesized by dissolving 4.84 g (0.01 mol) of Bi (NO_3_)_3_·5H_2_O in 25 mL of acetic acid solution (acetic acid: H_2_O 2:1 v/v) with stirring for complete dissolution. Next, 3.2 g (0.01 mol) of CTAC was dissolved in 25 mL of deionized water and added to the nitrate solution. The resulting solution was stirred for 30 min. The pH was then adjusted to 13 using a 10 M NaOH solution. After an additional 30 min of stirring, the reaction mixture was subjected to microwave treatment (1000 W; 2450 Hz) for 10 min at atmospheric pressure. The resulting product was collected by centrifugation, washed several times with ethanol: water, and dried in an oven at 60 °C for 18 h.

#### Synthesis of self-assembled rod-shaped porphyrin

Aggregated rod-shaped 5,10,15,20-Tetrakis (4-carboxyphenyl) porphyrin was synthesized by an acid–base neutralization self-assembly strategy, in the presence of CTAB surfactant [26]. In a typical synthesis, 0.04 g of porphyrin powder was dissolved in 0.5 mL of 0.2 M NaOH (referred to as the host solution). The “guest’ solution was prepared by dissolving 0.036 g of CTAB in 10 mL of 0.01 M HCl. The guest solution was quickly injected into the host solution with vigorous stirring for 30 min in the dark. The resulting precipitate was collected by centrifugation at 9,000 rpm for 10 min and washed with deionized water several times to remove the surfactant.

In the preparation of aggregated rods porphyrin, the monomeric porphyrin molecules were assembled through a CTAB-assisted process. The assembly was promoted by the hydrophobic interaction between the alkyl group of CTAB and the porphyrin molecule [36] as well as the transformation from the H-aggregates to the J-aggregates mode [36]. The J-aggregation mode, obtained a lower pH (~ 3) medium demonstrates a superior photocatalytic activity compared to the H-aggregates [26]. The π-π and hydrogen bonding interactions in J-aggregation promote coherent electron delocalization, charge transfer and separation which enhances their performance. It is also speculated that the rapid formation of the aggregated form is spurred by the condensation reaction between the carboxyl substituent of the porphyrin and the amino group of CTAB [20].

#### Synthesis of porphyrin@Bi_12_O_17_Cl_2_

The preparation of porphyrin@Bi_12_O_17_Cl_2_ containing 0.02% wt., 0.1% wt., 0.4% wt., 1% wt., and 10% wt., porphyrin was carried out as follows: 0.2 g of as-synthesized BOC was added to 20 mL of methanol containing a certain amount of Porph (0.04 mg, 0.2 mg, 0.8 mg, 2 mg, and 20 mg). The mixture was sonicated for 5 min and then stirred at 60 °C in the dark to completely volatilize the methanol. Since Porph is insoluble in water but organic solvent [37], the coupling of Porph to BOC to form Porph@BOC was performed in methanol. The coupling reaction involved the carboxylic (-COOH-) and hydroxyl (OH-) groups from the Porph molecules and BOC material. This interaction results in a strong ester bond that ensures tight composition between the organic and inorganic material. The resulting materials were collected and washed with water to remove unattached Porph and were labeled as *x*%Porph@BOC (0.02%Porph@BOC, 0.1%Porph@BOC, 0.4%Porph@BOC, 1%Porph@ BOC and 10%Porph@BOC), where *x* represents the weight percentage of Porph in BOC.

### Characterization

X-ray diffraction (XRD) was analyzed using a PANalytical X'Pert Pro powder diffractometer in θ–θ configuration with an X'Celerator detector and variable divergence and fixed receiving slits. The X-ray source used was Co-Kα radiation (λ = 1.789 Å) with Fe filtering. Fourier-transform infrared spectroscopy (FTIR) spectra were collected using a Varian FT-IR spectroscopy in a range of 500 and 4000 cm^−1^. Raman Spectroscopy (RAMAN) measurements were performed using a WITec alpha-300 RAS + Confocal micro-Raman microscope (Focus Innovations, Ulm Germany). Ultraviolet–Visible Diffuse Reflectance Spectroscopy (UV–Vis DRS) was measured on a Spectrophotometer U-3900 (Hitachi, Japan) in the range of 250 nm and 900 nm. Brunauer–Emmett–Teller (BET) specific surface areas measurements was performed on NOVA touch 2LX and the pore size was calculated from desorption isotherm via a density function theory (DFT) software, as part of equipment analysis system. Scanning electron microscopy (SEM) images were captured with Zeiss Ultra PLUS FEG-SEM (Ashikima Shi, Japan), energy-dispersive spectroscopy (EDX) analyser was combined with the scanning module. Electrochemical Impedance Spectroscopy (EIS) was measured on a multi-channel Biologic VMP-300 potentiostat (Knoxville TN37,930, USA) in the frequency range of 100 kHz – 10 mHz with an amplitude of 10 mV. Photoluminescence (PL) measurement was measure on Spectro Fluorophotometer RF-600 (Shimadzu, Japan) at an excitation wavelength of 350 nm, and the emission was scanned between 200 and 900 nm. RhB dye degradation was monitored by UV-16000PC Spectrophotometer (VWR). Electrochemical impedance spectroscopy (EIS) was measure in by VMP300 Bio-Logic potentiostat (Knoxville TN, USA).

### Degradation studies

Degradation studies were conducted using RhB dye as a representative contaminant. 60 mL of 20 mg L^−1^ of RhB dye was placed in a 100 mL-beaker reactor, and 0.025 g of photocatalyst material was added to the solution. The mixture was stirred in the dark for 3 h to achieve adsorption–desorption equilibrium. The solution was then irradiated for 4 h 30 min with visible light from six 16W fluorescent tubes (OSRAM, Germany) having a total intensity of 6300 lux. To monitor the degradation of the RhB dye, 2 ml of aliquots were withdrawn at 0.5 h intervals. The photocatalyst particles were separated using a centrifuge and the absorbance were measured with a spectrophotometer at 553 nm. The concentration of RhB dye was calculated from the equation of the standard curved. Photolysis experiments were also performed in the absence of photocatalyst. The percentage degradation was calculated from the following equation:

$$\frac{Co-Ct}{Co} \mathrm{x }100\mathrm{\%}$$

where *C*_*0*_ and *C*_*t*_ are the initial concentration and concentration at time of RhB dye.

### Recyclability test

The recyclability test was conducted using the 1%Porph@BOC material according to the method described in Sect. "Degradation Studies". Four runs were performed in the recyclability test, and after each run, the material was recovered, thoroughly washed with water and ethanol, and reused in subsequent steps.

### Radical Trapping Experiment

Radical trapping experiment was performed to determine the reactive oxygen species involved in the photocatalytic degradation of RhB dye. The scavengers used in the trapping experiments were EDTA-2Na (as h^+^ quencher), 1,4-benzoquinone (BQ) (as O_2_∙^−^ quencher), isopropanol alcohol (IPA) (as OH^∙^ quencher) and AgNO_3_ (as e^−^ quencher). In conducting the scavenging experiment, a scavenger was added to a mixture of RhB dye solution (20 mg L^−1^) and photocatalyst material (0.025 g) this gave a final concentration of a scavenger in the reaction mixture as 2.0 mmol·L^−1^. The mixture was irradiated with visible light to initiate the photocatalytic reaction and the analysis of RhB dye degradation was followed as described in Sect. "Degradation Studies".

## Results and discussion

### X-Ray diffraction

**Fig. 1:**
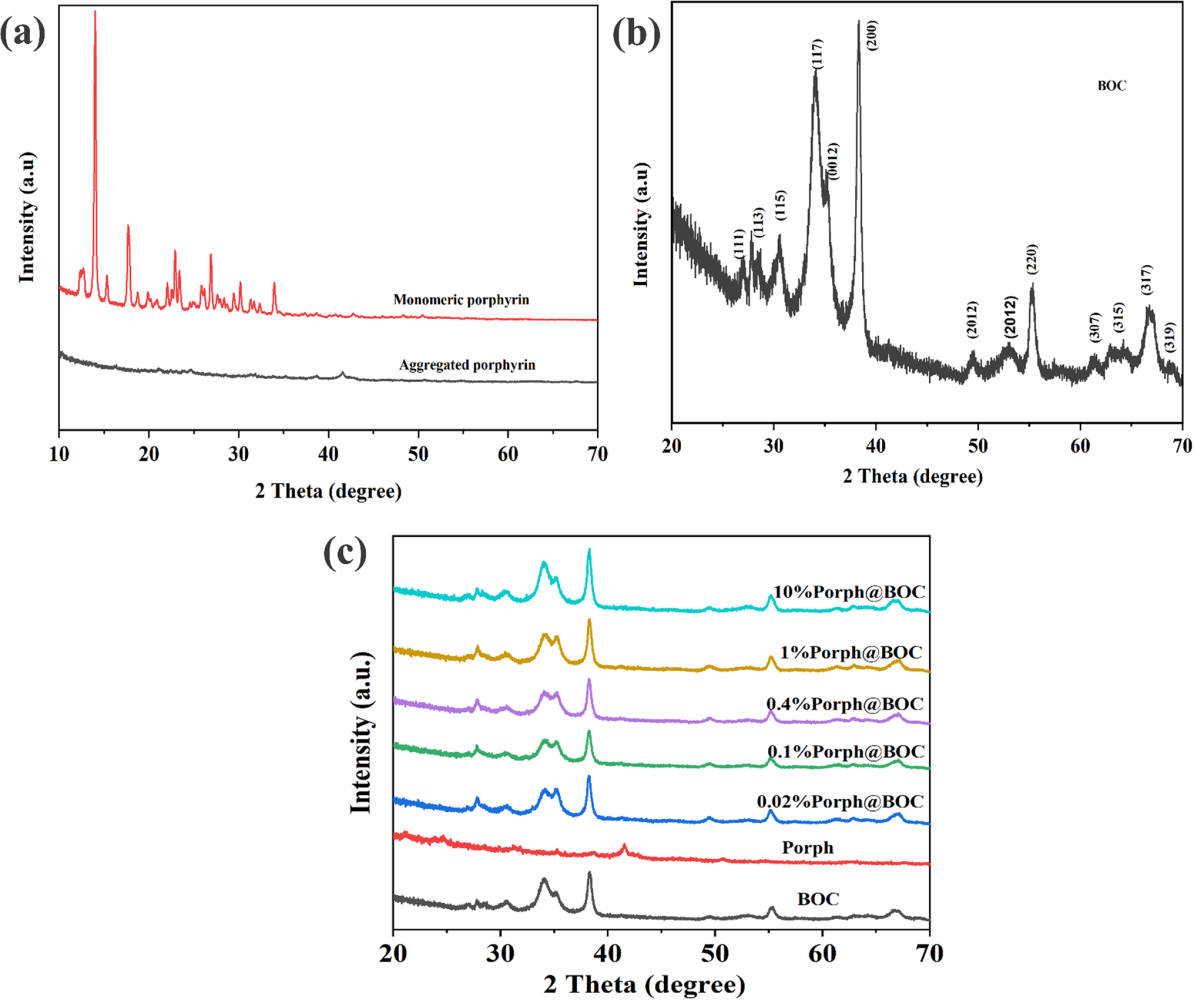
**a** XRD pattern of monomeric and aggregated porphyrin. **b** XRD peaks of pure BOC and **c** XRD patterns of different loading amounts of Porph in BOC

The XRD patterns of the monomeric and aggregated porphyrin are shown in Fig. 1a. The appearance of peaks in of the monomeric molecules demonstrated crystallinity while the weak peaks for aggregated porphyrin confirmed the nanostructured amorphous nature of the molecules [1]. Figure 1b showed the XRD pattern of pure Bi_12_O_17_Cl_2_. All the peaks from Bi_12_O_17_Cl_2_ sample matched those of diffraction planes of tetragonal Bi_12_O_17_Cl_2_ (JCPDS No. 37–0702) which are (111), (113), (115), (117), (0012), (200), (2012), (220), (315) (317) and (319) [1]. Figure 1c shows the XRD pattern of pure BOC, Porph and modified BOC samples (0.02%Porph @BOC, 0.1%Porph@BOC, 0.4%Porph @BOC, 1%Porph@BOC and 10%Porph@BOC). However, there was no appearance of peak attributed to the Porph phase in all modified samples due to the low concentration of Porph and amorphous nature of aggregated Porph.

### SEM and EDS

**Fig. 2:**
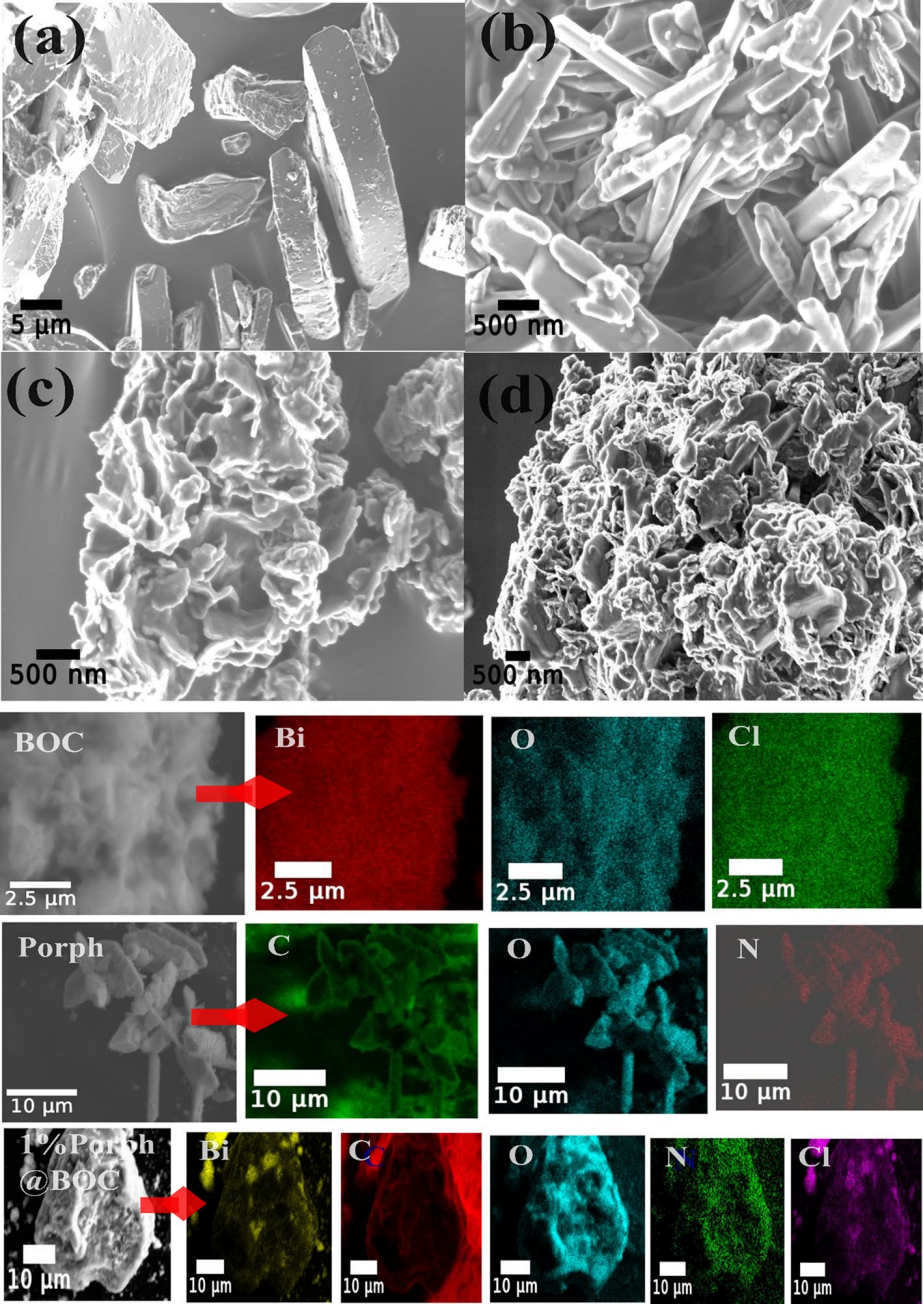
SEM image of **a** monomeric porphyrin. **b** aggregated rod porphyrin **c**; hierarchical flower-like BOC. **d** 1%Porph@BOC, followed by elemental mapping BOC, Porph and 1%Porph@BOC

Figure 2a-d display SEM images of monomeric porphyrin, aggregated porphyrin, BOC, 1%Porph@BOC. The monomeric porphyrin consists of block particles [38], while aggregated porphyrin was composed of rods generated from self-assembly during hydrophobic interaction between the alkyl group of the CTAB surfactant and porphyrin in the medium [36]. The BOC hierarchical structure was composed of numerous slabs subunit measuring about 500 nm. The aggregated porphyrin is composed of rods with majority of the rods ranging from 500 nm to 1 µm in length with average diameter of 50 nm as estimated from “Image J” software. Furthermore, EDS elemental mapping was conducted on BOC, Porph and 1%Porph@BOC, and it indicates the presence and uniform distribution of constituent elements Bi, O, Cl, N and C in the materials.

### Optical property and band gap energy

**Fig. 3:**
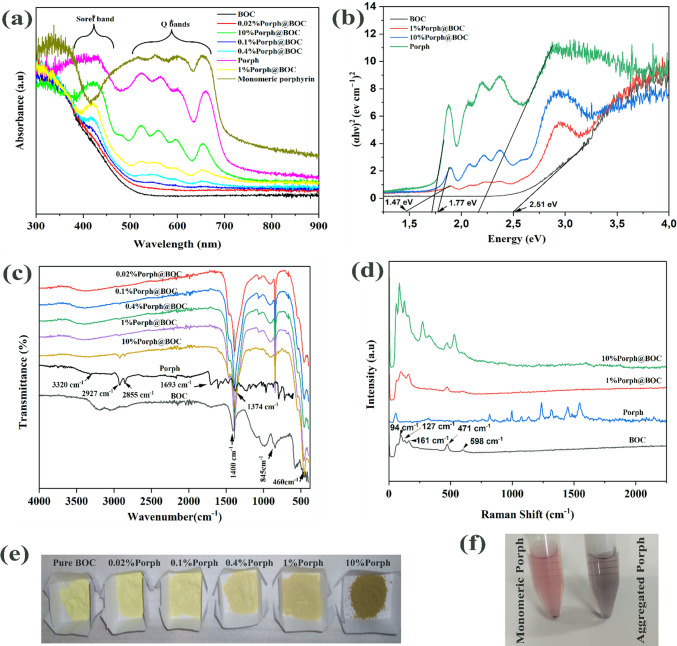
**a** UV–vis DRS of BOC, Porph, *x*%Porph@BOC **b** Tauc plot for band gap energy estimation for BOC, Porph, 1%Porph@BOC and 10%Porph@BOC. **c** FTIR spectra of *x*%Porph@BOC, Porph and BOC. **d** RAMAN spectra of 10%Porph@BOC, 1%Porph@BOC Porph and BOC **e** Varying Porph loading in *x*%Porph@BOC. **f** Monomeric Porph and aggregated Porph in methanol

Figure 3a shows the UV–Vis DRS measurement of the samples. Aggregated Porph show a very strong absorption with absorption edge at 730 nm while the absorption edge for BOC is 500 nm. It is evident that the addition of Porph, gradually increased visible light absorption of the composite.

The UV–vis DRS spectra, indicate a batochromatic shift in the Soret band and the appearance of a strong Q band in the 660 nm for aggregated porphyrin, compared to 655 nm of monomeric porphyrin. Additionally, there is a red shift of the Soret band from 340 nm in monomeric porphyrin to 421 nm in aggregated Porph, indicating J-form aggregation [39] The shift in the band for all other sample loadings with Porph compared with Porph can be attributed the interaction between the Porph and BOC [40].

The presence of characteristic Soret and Q bands for all samples, although weak in samples with low porphyrin content, indicates the presence of Porph the samples. Therefore, the UV–vis DRS confirmed the formation of a close bond between anchoring groups of Porph with BOC, as indicated by the intense optical density and the red shift in the Soret band [41].

From the UV–Vis DRS data, the band gap energy of Porph, BOC, 1%Porph@BOC and 10%Porph@BOC were estimated in Fig. 3b. The band gap energy was calculated from the Tauc plot, *αh* = *a*(*hv* − *E*_*g*_)^n/2^ where *α,* h*, v, E*_*g*_ are the adsorption coefficient, Plank constant, frequency of light and band gap energy, respectively. The value of *n* is 1 for direct transition and 4 for indirect transition. The BOC and Porph samples exhibit direct type transition [42] so *n* takes the value of 4. The band gap energy of BOC, 1%Porph@BOC, 10%Porph@BOC and Porph were estimated to be 2.51 eV, 1.47 eV, 1.77 eV and 1.71 eV, respectively. The presence of Porph in the composite material reduces the band gap energy and enhances the light adsorption function the materials [35]. In Fig. 3e, the colour of the *x*@%Porph@BOC is shown, the yellow colour of BOC becoming darker as Porph content is increased. The colors of modified porphyrin (aggregated) and unmodified porphyrin (monomeric) in methanol are displayed Fig. 3f, showing the monomeric porphyrin as purple while the aggregated porphyrin shows a darker purple hue.

### FTIR and RAMAN spectroscopy

Figure 3c, d show the FTIR and RAMAN spectra of BOC, Porph and *x%*Porph@BOC to determine functional groups and structure of materials. The characteristic FTIR peaks of B_12_O_17_Cl_2_ appearing at ~ 460 cm^−1^, 846 cm^−1^, and 1400 cm^−1^ are attributed to stretching vibrations of B-O, bending vibration of O-Bi-O and the asymmetric stretching vibration of Bi-Cl [43]. Characteristics peaks of Porph can be observed at ~ 1374 cm^−1^, ~ 1693 cm^−1^ ~ 3320 cm^−1^ are due to vibration of N–H bond. In addition, peaks at ~ 2927 cm^−1^ and ~ 2850 cm^−1^ present are assigned to asymmetric and symmetric stretching vibrations of C-CH_2_ in Porph [32]. The ~ 2927 cm^−1^ and ~ 2850 cm^−1^ peaks were present in 10*%*Porph@BOC which confirms the presence of Porph in the materials. However, these peaks were absent in other *x%*Porph@BOC materials due to low loading of Porph. Also, the absence of Porph peaks in these samples may be due to the presence of inorganic BOC that can decreases the peak intensity, resulting in the non-detection of porphyrin peaks in the organic–inorganic composite [32]. However, UV–Vis DRS measurement in Fig. 3a confirmed the presence of Porph, from the Soret and Q bands, thus confirming the successful formation of *x%*Porph@BOC composites.

In the Raman measurement, 94 cm^−1^, 127 cm^−1^, 161 cm^−1^, 471 cm^−1^ and 598 cm^−1^ were observed which are all characteristics peaks of pure Bi_12_O_17_Cl_2_ [6, 44]. The peak located at 94 cm^−1^, 127 cm^−1^ and 161 cm^−1^ are assigned to external Bi-Cl stretching mode of E_g_ and A1_g_, respectively. The peak at 471 cm^−1^ is assigned to the B1_g_ motion of oxygen atoms [45]. The vibrational peaks recorded for Porph are 997 cm^−1^, 1237 cm^−1^, 1320 cm^−1^_,_1450 cm^−1^,1547 cm^−1^ and are close to vibration peaks previously reported for C_α_–C_m_ (1004 cm^–1^ and 1552 cm^–1^), C_α_–N (1242 cm^–1^), C_α_–C_β_ + C_β_–H (1330 cm^–1^ and 1457 cm^–1^), and the weak peak at 1497 cm^–1^ [37]. From the Raman results, there were no significant changes in the structure of BOC material when Porph was loaded .

### Photocatalytic degradation experiment

**Fig. 4:**
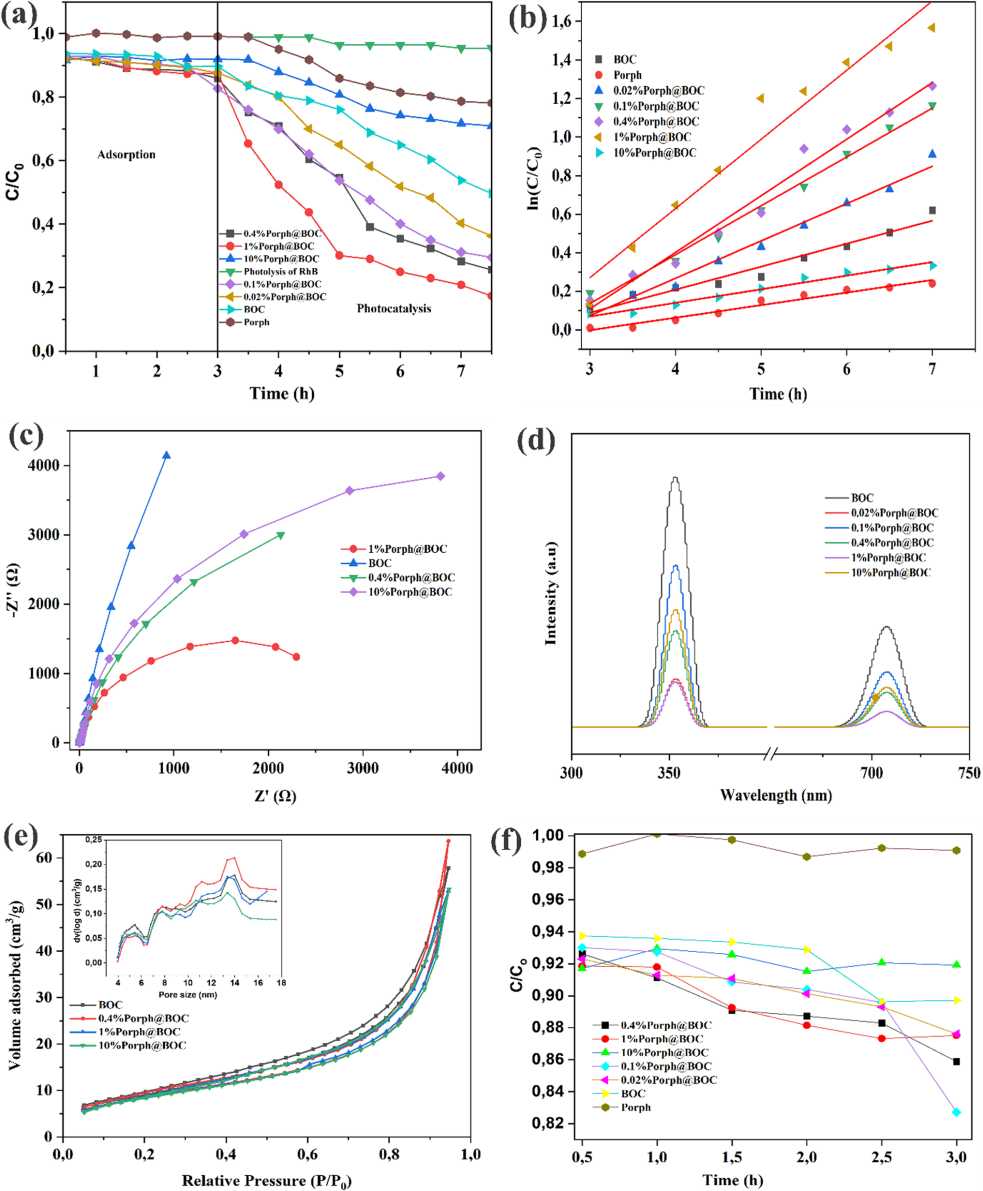
**a)**Photocatalytic degradation of RhB dye on *x*%Porph@BOC. **b** Rate constants of Photocatalytic degradation of RhB dye on *x*%Porph@BOC materials. **c** EIS measurement of BOC, Porph and *x*%Porph@BOC **d** Photoluminescence spectra of BOC, Porph and *x*%Porph@BOC **e** N_2_ adsorption desorption isotherm. inset: Pore size distribution of selected *x*%Porph@Bi_12_O_17_Cl_2_ and **f** Adsorption of RhB dye on x%Porph@Bi_12_O_17_Cl_2_ materials

The results of the photocatalytic degradation of RhB dye on the prepared materials is shown in Fig. 4a. The reaction mixture was stirred in the dark for 180 min before light was irradiated to start the photocatalytic reaction. The photocatalytic degradation efficiency was determined from the following equation.

$$\frac{Co-Ct}{Co} \mathrm{x }100\mathrm{\%}$$

where *C*_*0*_ and *C*_*t*_ are the initial concentration and concentration at time of Rh B dye.

From Fig. 4a, after 60 min of irradiation of light, the degradation efficiency of RhB dye degradation on BOC, Porph, 0.02%Porph@BOC, 0.1%Porph@BOC, 0.4%Porph@BOC, 1%Porph@BOC and 10%Porph@BOC were reported to be 31.1%, 16.5%, 41.8%, 52.4%, 60.9%, 71% and 23.6%, respectively. Among the modified materials, 1%Porph@BOC performed the best with an efficiency of 71% while, 10%Porph@BOC was the least performing material with an efficiency of 23.6% efficiency, which is less than that of BOC with an efficiency of 31.1%. At the end of the photocatalytic degradation experiment (4.5 h), 82.6% degradation efficiency was achieved on 1%Porph@BOC. It was also demonstrated that photolysis did not contribute significantly to the removal of RhB dye and only achieved a removal efficiency of efficiency of 3.5%.

The kinetics of RhB dye degradation was determined from the data obtained in the degradation study to be pseudo-first order kinetic equation. First-order kinetic –in C/C_o_ = kt where C_0_ and C are the initial and final concentrations of Rh B dye, *k*, the rate constant, and *t*, the time, was used to fit the experimental data. The pseudo-first kinetic was confirmed from the linear curve in Fig. 4 b. The R^2^ values for 0.02%Porph@BOC, 0.1%Porph@BOC, 0.4%Porph@BOC, 1%Porph-@BOC, 10%Porph@BOC and BOC were found to be 0.9566, 0.97107, 0.97808, 0.99063, 0.97548, 0.94701, 0.97018, respectively, as presented in Table 1. Overall, the degradation of RhB dye on 1%Porph@BOC was 3.1 times faster than on pristine BOC.

**Table 1:** First order reaction constant k for the %Porph@BOC materials

Material	k (h^−1^)	R^2^
BOC	0.12	0.96
Porph	0.065	0.97
0.02%Porp@BOC	0.19	0.98
0.1%Porp@BOC	0.25	0.99
0.4%Porp@BOC	0.29	0.98
1%Porp@BOC	0.36	0.95
10%Porp@BOC	0.07	0.97

### The role of porph in the photocatalysis of *x*%Porph@BOC

The photocatalytic behavior of porphyrin in generating charge carriers and their separation when irradiated with light energy depends on whether they are free-standing or immobilized onto a support [46]. A support may not play a participatory role in the overall photocatalysis, as in the case of the free form [17]. Therefore, the type of support whether it actively participates in photocatalysis or not is crucial to the activity of the materials. In this study, BOC serves as a support and promoter [35], as well as a photocatalyst, giving rise to the novel materials *x*%Por@BOC, possessing different photocatalytic abilities than individual Porph and BOC [47]. Several factors contribute to the overall efficiency of photocatalysis of the *x*%Por@BOC materials namely photosensitization, surface phenomenon, material band gap modulation and heterojunction formation and interplay between these effects.

The light harvesting ability of the composite has been demonstrated earlier by the UV–vis DRS measurement (Fig. 3a). The visible-light harvesting properties of BOC was further strengthened by the incorporation of Porph, by extending the light absorption beyond 500 nm of BOC to ca. 750 nm of the composites. The improved visible light-harvesting ability of the *x*%Por@BOC translates into more photogenerated charge carrier and ultimately higher photocatalytic power. Additionally, from the Tauc plot (Fig. 3b), the band gap energy decreased from 2.51 eV for BOC to 1.47 eV for 1%Por@BOC by anchoring Porph to BOC.

Separation of photogenerated charge carriers is another factor that affects photocatalytic degradation ability of materials. Photoluminescence (PL) measurements and electrochemical impedance spectroscopy (EIS) studies reveal that the charge separation in the composite system was enhanced with the introduction of porphyrin. The arc radius of the EIS Nyquist plots spectra represents charge carrier separation corresponding to the photocurrent output. An increased radius of spectra indicates poor of electrons and holes separation efficiency, resulting in lower photocatalytic activity of the material [48]. EIS test was performed on 1%Porph@BOC, 0.4%Porph@BOC, 10%Porph@BOC, and BOC materials and shown in Fig. 4c. The 1%Porph@BOC material displays a lower semi-circle than the other materials [49], indicating enhanced charge transfer and reduced recombination. Photoluminescence (PL) measurement was also performed to provide information about the efficiency of photoinduced processes such as charge injection, transfer, and recombination. A weak emission spectrum indicates favorable photocatalysis as it demonstrates suppressed recombination of charge carriers by efficient transport of electrons and holes. Two emission spectra were observed at 709 nm [50] and 350 nm [43], as presented in Fig. 4d. The lowest luminescence was observed with 1%Porph@BOC compared to BOC and 10%Porph@BOC which indicate suppressed recombination of photogenerated electrons and holes in the system.

The photocatalytic degradation occurs on the surface and is influenced by surface area which bears active sites of the materials. The contribution of surface area to photocatalysis was supported by the adsorption studies of RhB dye on BOC (Fig. 4f). The increased Porph content in the composite decreased the available surface and active sites for adsorption of the pollutant for photodegradation [51]. The poor adsorption of pollutant to aggregated Porph-laden 10%Porph@BOC blocked the active sites of BOC, leading to the deterioration of photocatalytic efficiency [46]. The adsorption curve of 10%Porph@BOC was rather erratic showing constant adsorption, unlike for 0.02%Porph@BOC, 0.1%Porph@BOC, 0.4%Porph@ BOC and 1%Porph@BOC, which showed increased in adsorption of the pollutant over time. The adsorption observed for 10%Porph@BOC and Porph standalone share similar characteristics while that of 0.02%Porph@BOC, 0.1%Porph@BOC, 0.4%Porph@BOC and 1%Porph@BOC share similarity which points to the effect of Porph on the adsorption of pollutant onto the composite. Although the introduction of more Porph in BOC enhances light adsorption, the interplay with surface area and active site does not guarantee a linear relationship with photocatalysis. The study established an optimum loading amount of Porph in the composite and found out that balancing of surface-active sites of BOC and light trapping by Porph is essential for optimum performance of the hybrid material. The synergy between the Porph and BOC lies in the fact that Porph induces high oxidation of electrons and holes by facilitating shuttling, transfer, and separation in the heterostructure, while BOC provides the active sites for reaction.

**Fig. 5:**
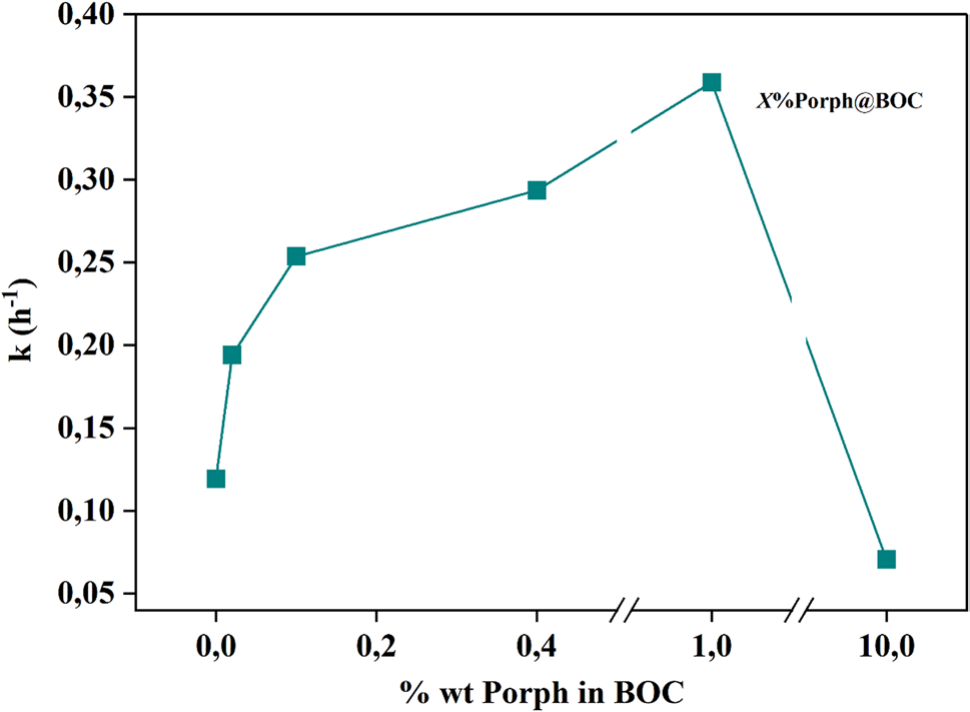
Percentage weight of aggregate porphyrin, x, in BOC versus kinetic rate constant, k, of degradation of RhB dye

In Fig. 5, the photocatalytic degradation of RhB dye on the materials shows a gradual increase in the following order: 0.02%Porph@BOC < 0.1%Porph@BOC < 0.4%Porph@BOC < 1%Porph@BOC. At 10%Porph@BOC, there was a sharp decrease in the photocatalytic activity, attributed to the reduction in the surface area of BOC from 31.7 m^2^/g to 30.8 m^2^/g and a decrease in the number of active sites on BOC (Table 2). The specific surface areas and the pore sizes of the material were not correlated. The N_2_ adsorption–desorption isotherms and the corresponding pore size distribution curves of selected materials are shown in Fig. 4e. The samples isotherm can be classified as Type IV curves with type 3 hysteresis which points to the presence of mesoporous structure.

**Table 2:** Surface BET analysis

Material	S_BET_ Surface Area (m^2^/g)	Average particle diameter (nm)	Average pore diameter (nm)
BOC	31.72	85.98	11.31
0.4%Porph	30.10	90.60	13.11
1%Porph	29.75	91.68	11.02
10%Porph	30.84	88.42	10.07

### Radical trapping experiment

**Fig. 6:**
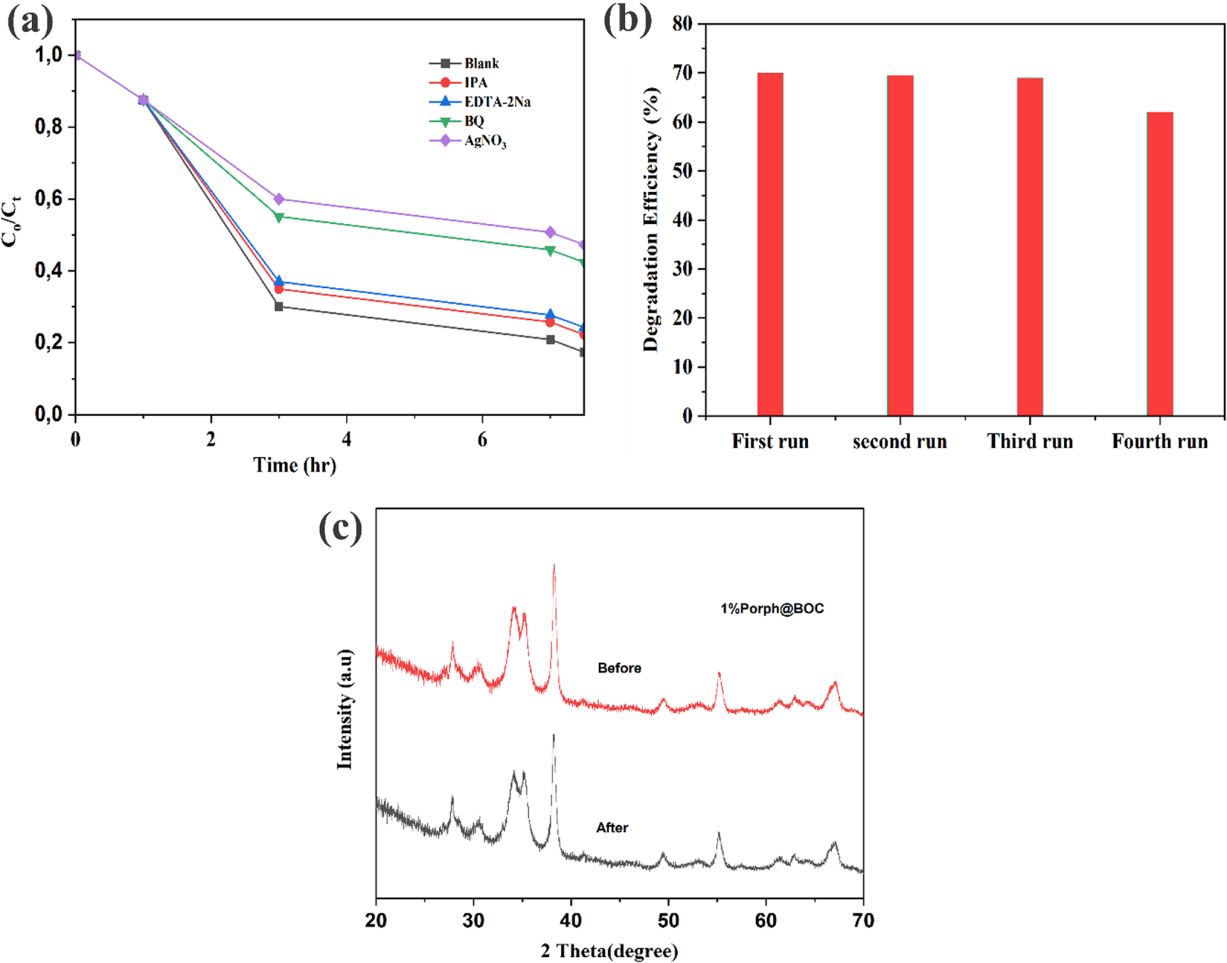
**a** Effect of various scavengers on degradation efficiency** b** Recyclability tests: Efficiency as a function of degradation run **c** XRD peak of 1%Porph@BOC before and after the fourth run

The reactive species involved in photocatalytic degradation are the electrons (e^−^), holes (h^+^), superoxide (O_2_^∙−^) and hydroxyl radical (OH^∙^). These species are either generated as primary or secondary species when a photocatalyst material is activated by light. Electrons and holes are generated as primary species which subsequently gives rise to other secondary species as shown in the equations in Sect. 4. To determine the reactive species involved in the photocatalytic degradation of RhB dye, scavenging tests were performed applying the following scavengers: EDTA-2Na (as h^+^ quencher); 1,4-benzoquinone (BQ) (as O_2_^∙−^ quencher), isopropanol alcohol (IPA) (as OH^∙^ quencher) and AgNO_3_ (as e^−^ quencher). Figure 6a shows the effect of the various scavengers on degradation efficiency. The dominant species were e^−^ and O_2_∙^−^, reducing the photocalytic efficiency from 70 to 60% and 55%, respectively, when the respective scavangers were applied. The degradation efficiency was reduced by 7% and 5% to 63% and 65%, respectively, in the presence of BQ and IPA, demonstrating that the OH^∙^ and h^+^ play a lesser role in the degradation process.

The recyclability of the material was tested to determine the suitability of the composite in photocatalysis. Reuse of the material will largely depend on the resistance of the composite material to photo-corrosion. Continuous exposure of photocatalyst materials in mostly aqueous medium could result in the alteration of the crystallinity, photo-corrosion and other adverse changes, which could negatively affect their photocatalytic activity. From the results presented and discussed thus far, Porph on BOC improved photocatalytic efficiency, and the 1%Porph@BOC demonstrates optimum performance. Four runs were performed in the recyclability test and for each run, photocatalyst materials are recovered from previous step, washed, and reused for subsequent cycles. An average of 90% of the photocatalyst was recovered at each cycle. Due to the loss of photocatalyst material at every cycle, the volume of RhB dye was adjusted accordingly such that for every cycle the “0.025 g photocatalyst/60 ml of RhB dye” (See Sect. "Degradation Studies"), was kept constant throughout. Figure 6b shows the degradation efficiency over the four cycles. After the first two runs, the photocatalytic efficiency was 70% and 69.5%. In the third and fourth runs, 65% and 60% efficiency were recorded. At the end of the fourth run, a photocatalytic efficiency retention of 85.7% was achieved which also meant that the integrity of Porph@BOC was largely preserved. Since Porph on the material contributes significantly to its efficiency, it can be argued that the loss of Porph in the photocatalytic process was minimal. This also demonstrates the tight ester bond formed between the carboxyl group of Porph molecules and the hydroxyl group on BOC [41]. The composite was shielded from photo-corrosion because of the aggregated form of Porph. Furthermore, it was demonstrated from the XRD pattern of 1%Porph@BOC before and after the fourth run that the material retained its crystal structure (Fig. 6c).

#### Mechanism of photocatalysis

**Fig. 7:**
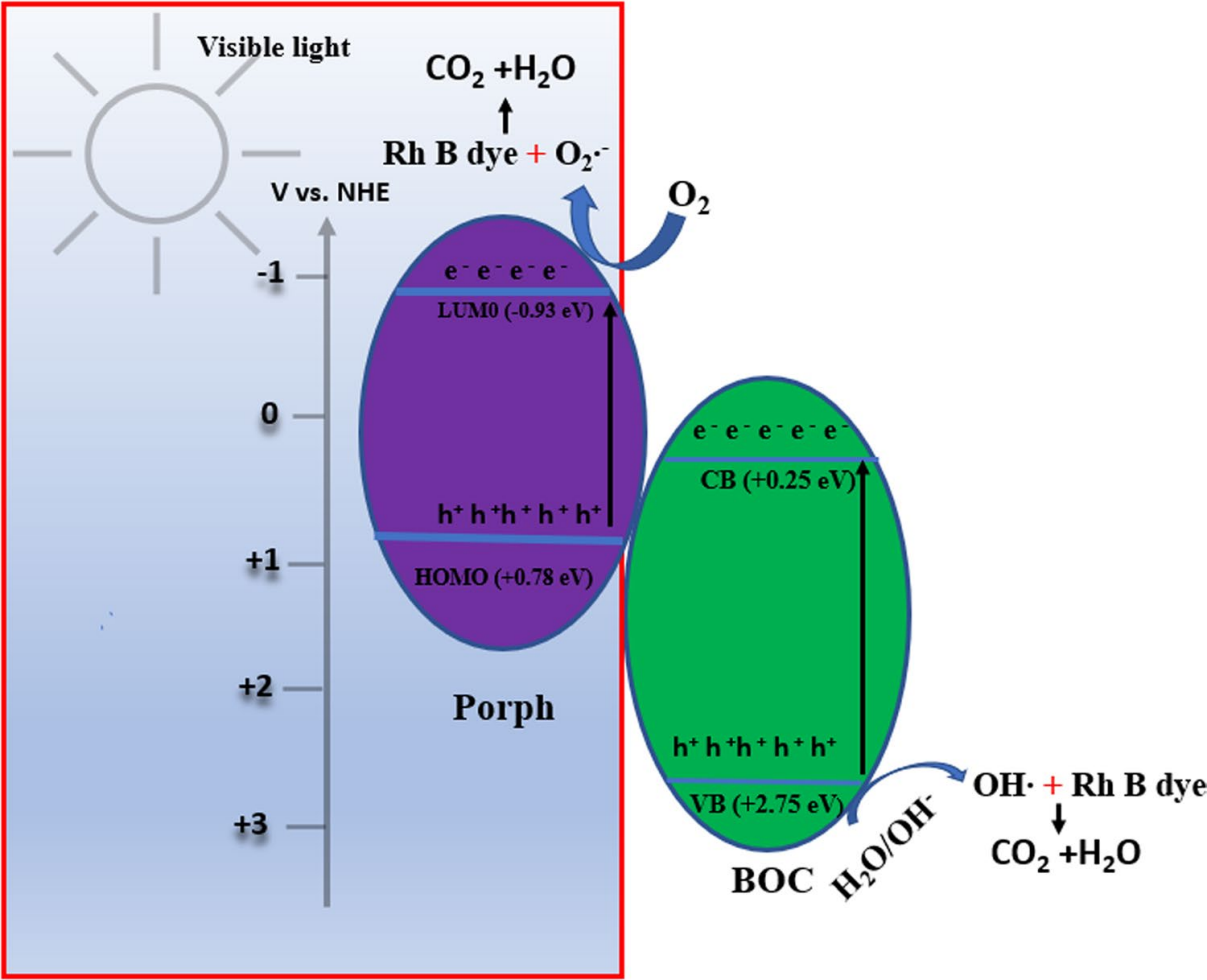
Band structures and photocatalytic mechanism of RhB dye degradation on Porph@BOC C

From the active species involved, conduction band (CB) and valence band (VB) position, band gap energy (E_g_), the potential diagram was constructed and a plausible mechanism for the degradation process (Fig. 7). The degradation of RhB dye is primarily facilitated by e^−^ and O_2_∙^−^, and to a lesser degree, h^+^. The e^−^ is first generated by the incident light photons and in turn generate O_2_^**∙−**^ (Eq. 5). The band gap energy of 1%Porph@BOC of the composite was estimated to be 1.77 eV. Additionally, the respective band gap energy of Porph and BOC were determined to be 1.71 eV and 2.51 eV, respectively. The CB and VB positions of BOC were determined using the following formula:

1 $$E_{{VB~~~~}} = {\text{ }}X{\text{ }} - {\text{ }}Ec + {\text{ }}0.5E_{g}$$

2 $$E_{{CB~~~~~}} = {\text{ }}E_{{VB}} - {\text{ }}E_{g}$$

where *E*_*VB*_ and *E*_*CB*_ are the valence and conduction band edge potentials, respectively, X is the Mulliken’s electronegativity which is 6 eV for BOC [6], E_c_ is the energy of free electrons on the hydrogen scale (~ 4.5 eV), and E_g_ is the band gap energy of corresponding semiconductors (BOC: 2.51 eV). Thus, *E*_*VB*_ and *E*_*CB*_ for BOC were calculated as 2.75 eV and 0.24 eV, respectively. Previous study reported the LUMO and HOMO of porphyrin to be − 1.00 eV and + 0.85 eV (vs NHE), respectively [52] and the band gap as 1.85 eV. The band gap determined from this study is 1.77 eV and the HOMO and LUMO were appropriately determined to be 0.78 eV and 0.93 eV, respectively.

Prior to contact, Porph with BOC form a staggard band structures (Fig. 7). Upon contact, e^−^ flow from Porph to BOC due to the higher work function of BOC. During this process, Porph and BOC reach equilibrium in terms of Fermi levels (E_f_) [53]. At the junction between the materials, BOC becomes negatively charged and Porph, positively charged. This leads to energy band bending and creation of an internal electric field (IEF) at the interface, with direction pointing from Porph to BOC.

The CB potential of BOC is not negative enough to react with O_2_ to produce O_2_∙^−^. Since the oxidation–reduction potential of O_2_/O_2_∙^−^ couple is -0.33 eV. It is expected that the more negative the LUMO e^–^ (-0.93 eV) can convert O_2_ to O_2_∙^−^. On the other hand, the BOC h^+^ (2.75 eV) is capable of oxidizing OH^−^ to OH∙ (OH^−^/OH∙; + 2.38 eV). The traditional pathway where e- move from Porph to the CB of BOC and the h^+^ from BOC to VB of Porph does not align with the mechanism involving O_2_∙^−^ and OH^−^ in the degradation of RhB dye. The redox potential of the accumulated e^–^ and h^+^ in the CB_LUMO_ and VB_BOC_ are not sufficient, as they are lower than the redox potentials of O_2_/O_2_∙^−^ and OH^−^/OH couples. To explain the high reduction potentials of the e^−^ and h^+^, the Step-(S-scheme), was employed to understand the photocatalytic process. In the S-Scheme, high redox e^–^ and h^+^ are generated and preserved while the ineffective ones are recombined and eliminated [54].

When the material is exposed to visible light, it absorbs photon energy because the band gap energy is lower than the photon energy of incident light. As a result, the e^−^ are separated from the HOMO of Porph and VB of BOC and they accumulate in the LUMO and CB. The e^−^ from CB of BOC, propelled by the bent energy band and the IEF, flow into Porph and recombine with HOMO’s h^+^. This ensures the preservation of high redox LUMO e^−^, while the unnecessary photogenerated charge carriers recombined and are eliminated. This Step-(S-scheme), improves the lifetime of charge carriers, thereby enhancing the degradation of a contaminant. The e^−^ in the LUMO of Porph reduce O_2_ in the water medium to O_2_∙^−^ which in turn oxidizes the RhB dye. Additionally, the h^+^ in the VB of BOC generate OH^∙^ from H_2_O and OH. The Porph is regarded as an e^−^ pump.

The following equations summarize the involvement of the various species in the degradation process.

3 $${\mathbf{BOC}} + hv\left( {{\mathbf{vis}}} \right) \to {\mathbf{BOC}}^{{*}}{\text{ }}\left( {{\mathbf{e}}_{{{\mathbf{CB}}}} ^{{-}} +{\mathbf{h}}_{{{\mathbf{VB}}}} ^{ + } } \right)$$

4 $${\mathbf{Porph}}{\mkern 1mu} + hv\left( {{\mathbf{vis}}} \right) \to {\mathbf{Porph}}^{{*}}{\text{ }}\left( {{\mathbf{e}}_{{{\mathbf{LUMO}}}} ^{{-}} +{\mathbf{h}}_{{{\mathbf{HOMO}}}} ^{ + } } \right)$$

5 $${\mathbf{H}}_{{\mathbf{2}}} {\mathbf{O}} \, + \, {\mathbf{h}}_{{{\mathbf{VB}}}}^{ + } \to_{ } {\mathbf{OH}}^{ . } + {\mathbf{H}}^{ + }$$

6 $${\mathbf{OH}}^{ - } +^{{}} {\mathbf{h}}_{{{\mathbf{VB}}}}^{ + } \to {\mathbf{OH}}^{ \cdot }$$

7 $${\mathbf{e}}_{{{\mathbf{CB}}^{ - } }} + {\mathbf{O}}_{{\mathbf{2}}} \to {\mathbf{O}}_{{{\mathbf{2}} \cdot }}{ - }$$

8 $${\mathbf{Rhodamine}}{\mkern 1mu} + {\mkern 1mu} ({\mathbf{OH}}^{ \cdot } ,{\mathbf{e}}_{{{\mathbf{LUMO}},^{ - } }} {\mathbf{O}}_{{\mathbf{2}}}^{{ \cdot - }} {\text{and}}{\mkern 1mu} {\mathbf{h}}_{{{\mathbf{VB}} ^+ }} ) \to {\mathbf{intermediates}} \to {\mathbf{H}}_{{\mathbf{2}}} {\mathbf{O}}{\mkern 1mu} + {\mkern 1mu} {\mathbf{CO}}_{{\mathbf{2}}}$$

## Conclusion

Rod-shaped porphyrin, which is a photosensitizer, was prepared and coupled to Bi_12_O_17_Cl_2_ to enhance its photocatalytic efficiency. The photocatalytic efficiency of the composite was tested at various porphyrin doses of 0.02%, 0.1%, 0.4% and 1%, and 10% w/w, for photocatalytic degradation of RhB dye. An optimum dose of 1% w/w porphyrin in the composite was establishedwhich is a 3.1– fold increase in degradation efficiency  compared to bare Bi_12_O_17_Cl_2_. This enhanced performance was attributed to the synergy between the anchored porphyrin and Bi_12_O_17_Cl_2._ However, at the highest dose of 10%w/w porphyrins, a deterioration in the photocatalytic activity was observed due to blockage of active sites on Bi_12_O_17_Cl_2_ surface. Two phenomena were found to be crucial to the photocatalytic activity of the composite: the surface area of Bi_12_O_17_Cl_2_ and photosensitization effect of porphyrin. It is important to strike a balance between these effects in the synergy between Porph and BOC in the composite in order to achieve optimum performance. The optimum activity achieved with 1% wt. porphyrin in the composite, provided adequate exposure of active sites on Bi_12_O_17_Cl_2_ surface for the photodegradation of RhB with the simultaneous benefit of the photosensitization effect of porphyrin. It was determined that holes and superoxide were the dominant species in the degradation process It was established that *x*%Porph@BOC are tightly bound composites with no observable detachment of the porphyrin during photocatalysis.

## Data Availability

The datasets generated during and or analyzed during the current study are available from the corresponding author on reasonable request.
